# Appropriateness of psychotropic medication use in a cohort of adolescents with intellectual disability in Queensland, Australia

**DOI:** 10.1192/bjo.2020.125

**Published:** 2020-11-17

**Authors:** Menghuan Song, Robert S. Ware, Tan N. Doan, Lyn McPherson, Julian N. Trollor, David Harley

**Affiliations:** Queensland Centre for Intellectual and Developmental Disability, Mater Research Institute-University of Queensland, Mater Hospitals, Queensland, Australia; Queensland Centre for Intellectual and Developmental Disability, Mater Research Institute-University of Queensland, Mater Hospitals; and Menzies Health Institute Queensland, Griffith University, Queensland, Australia; Department of Medicine at The Royal Melbourne Hospital, University of Melbourne, Victoria, Australia; Queensland Centre for Intellectual and Developmental Disability, Mater Research Institute-University of Queensland, Mater Hospitals; and Menzies Health Institute Queensland, Griffith University, Queensland, Australia; Department of Developmental Disability Neuropsychiatry, Faculty of Medicine, University of New South Wales, New South Wales, Australia; Queensland Centre for Intellectual and Developmental Disability, Mater Research Institute-University of Queensland, Mater Hospitals, Queensland, Australia

**Keywords:** Psychotropic drugs, intellectual disability, off-label use, inappropriate prescribing, adolescent

## Abstract

**Background:**

Psychotropic medications are sometimes used off-label and inappropriately. This may cause harm to adolescents with intellectual disability. However, few studies have analysed off-label or inappropriate prescribing to this group.

**Aims:**

To examine the appropriateness of psychotropic prescribing to adolescents with intellectual disability living in the community in south-east Queensland, Australia.

**Method:**

Off-label medication use was determined based on whether the recorded medical condition treated was approved by the Australian Therapeutic Goods Administration. Clinical appropriateness of medication use was determined based on published guidelines and clinical opinion of two authors who specialise in developmental disability medicine (J.N.T. and D.H.).

**Results:**

We followed 429 adolescents for a median of 4.2 years. A total of 107 participants (24.9%) were prescribed psychotropic medications on at least one occasion. Of these, 88 (82.2%) were prescribed their medication off-label or inappropriately at least once. Off-label or inappropriate use were most commonly associated with challenging behaviours.

**Conclusions:**

Off-label or inappropriate use of psychotropic medications was common, especially for the management of challenging behaviours. Clinical decision-making accounts for individual patient factors and is made based on clinical experience as well as scientific evidence, whereas label indications are developed for regulatory purposes and, although appropriate at a population level, cannot encompass the foregoing considerations. Education for clinicians and other staff caring for people with intellectual disability, and a patient-centred approach to prescribing with involvement of families should encourage appropriate prescribing. The effect of the National Disability Insurance Scheme on the appropriateness of psychotropic medication prescribing should be investigated.

## Psychotropic medication use in people with intellectual disability

People with intellectual disability have significant limitations in intellectual functioning and adaptive behaviours.^1^ They often have co-occurring epilepsy or mental disorders including autism, and are likely to exhibit challenging behaviours.^2^ Psychotropic medications are frequently prescribed to manage these health problems.^3^ However, the use of psychotropic medications in people with intellectual disability carries risk.^4^

## Off-label use of psychotropic medications

Psychotropic medications are used off-label for managing challenging behaviours,^5,6^ but the evidence base is inadequate. Off-label use in Australia refers to the use of a registered medicine outside the product information approved by the Therapeutic Goods Administration (TGA).^7^ Medications are registered and approved for market entry by the TGA specifying indications, dose, route of administration and patient group.^8^ Off-label use lacks formal clinical trial evidence for effectiveness and safety.

## Clinically inappropriate use of psychotropic medications

The TGA focuses on market entry of medications, rather than the prescribing practices of clinicians. In clinical practice, medications are prescribed based on the knowledge and experience of clinicians. Clinically appropriate prescribing takes account of diagnosis, treatment plan (pharmacological or other approaches), drug choice, assessment of potential benefits and risks of the prescriptions, comorbidities and other health states of individual patients, and involves carers and patients in decision-making.

The use of psychotropics to manage challenging behaviours in the absence of mental disorders is sometimes inappropriate,^4^ and risk may outweigh benefit in these instances. Challenging behaviour is often precipitated by biological and psychosocial factors.^9^ Psychotropic medication use may be a restrictive practice when the drugs are used for their sedative effects, rather than for treating mental disorders. In Australia, reduction or elimination of restrictive practice has been recommended to protect the human rights and safety of people with disability.^10^ There is a lack of adequate evidence of effectiveness of psychotropic medications in managing challenging behaviours.^4^ Compared with the general population, people with intellectual disability may be more vulnerable to adverse effects associated with psychotropic medications, including weight gain, other metabolic effects and neurological symptoms.^11^

## Psychotropic medication use in adolescents with intellectual disability and study aims

Adolescents with intellectual disability are in a transitional stage that includes biological changes and cognitive and social adjustments.^12^ Aspects of this life stage, including mood and behavioural changes, may precipitate inappropriate prescribing of psychotropic medications. The prevalence of psychotropic medication use has been reported among Australian adolescents with intellectual disability,^13^ but there is insufficient data on appropriateness. Our study aims to investigate off-label and inappropriate use of psychotropic medication in adolescents with intellectual disability living in the community in south-east Queensland, Australia.

## Method

### Study design and participants

A cohort of adolescents with intellectual disability in south-east Queensland, Australia, was followed between January 2006 and June 2010. Data were originally collected as part of a randomised controlled trial, the Advocacy Skills Kit (ASK) study, designed to examine the usefulness of a health intervention package that includes a health check booklet and a hand-held health diary.^14–16^ All participants who had medical notes available for at least 3.5 years were included in this study. Participating adolescents lived in the community, had an intellectual disability, were aged between 10 and 20 years on 1 January 2006 and attended a special education school (SES) or special education unit (SEU). Adolescents were eligible to attend the SESs or SEUs only if they had been diagnosed with an intellectual disability by Education Queensland guidance officers or psychologists. Written individual consent was received from the adolescents’ carers and nominated general practitioners. The ASK trial was registered with ClinicalTrials.gov (NCT00519311). All procedures performed in this study involving human participants were in accordance with the ethical standards of the institutional committees and with the Helsinki Declaration of 1975, as revised in 2008. Ethical approval was granted by The University of Queensland Behavioural and Social Sciences Ethical Review Committee (approval number 2004000081) and the Queensland Government Department of Education and the Arts (approval number 550/27/424).

### Medications and medical conditions

Electronic or paper medical notes, including correspondence with specialists and investigation results, were collected on visits to participants’ general practitioners by our researchers. Medical notes were for the period from 1 January 2006 to the day of visit (between September 2009 and June 2010). Prescribed medications were extracted from the medical notes by an experienced nurse researcher. Medication categorisation was conducted by a pharmacist (M.S.) according to the anatomical therapeutic chemical classification system.^17^ Psychotropic medications were categorised as antipsychotics, antidepressants, anxiolytics, psychostimulants, hypnotics/sedatives and anti-Parkinsonian medications. When a psychotropic drug was prescribed, its generic name, dosage form (e.g. capsule, liquid), strength and conditions treated were recorded. Medical disorders were extracted from medical notes and were coded with the ICD-10.^1^ The codes were checked by an experienced specialist in adult developmental disability medicine. Challenging behaviours were behaviour related problems identified by clinicians and recorded in medical notes. They were classified with assistance from an experienced psychologist and included aggression, impulsivity, self-injury, withdrawal, property destruction, sexually inappropriate behaviour, socially inappropriate behaviours (fixated and repetitive behaviour, hyperactivity, agitation, non-cooperation, restlessness, exposing body inappropriately, lying, stealing, swearing, screaming, deliberately vomiting, stalking people, threatening people, echolalia, coprophagia and laughing inappropriately), other specified behaviours (obsession, absence, poor concentration span and non-adherence to drugs), non-specified, and multiple behaviours (two or more concurrent behaviours). The presence of mental disorder and challenging behaviour varied through the study. Autism and attention-deficit hyperactivity disorder (ADHD) were assumed to be chronic for study purposes.

Use of psychotropic medications was examined in terms of label adherence and appropriateness. Label adherence (on- or off-label) was determined based on whether the recorded medical condition treated was present in the TGA-approved indications. Assessment of appropriateness was performed independently by a general practitioner (D.H.) and a neuropsychiatrist (J.N.T.). There is no specialist training pathway for developmental disability medicine in Australia, but authors D.H. and J.N.T. are both experienced specialists in clinical management of people with intellectual disabilities, particularly in managing challenging behaviour. In determining clinical appropriateness for presentations other than challenging behaviour, diagnoses from medical notes were scrutinised, and widely used clinical guides, particularly The Therapeutic Guidelines ‘eTG’, were consulted.^18^ When deciding clinical appropriateness for challenging behaviours, medical notes were inspected and non-drug approaches and nature of challenging behaviour were assessed. D.H. and J.N.T determined the condition for which medication had been prescribed, and applied a consistent and defensible set of thresholds to define appropriate and inappropriate prescribing of psychotropic medications for challenging behaviour. A systematic process was independently followed as two steps. In step 1, decisions were made for whether the challenging behaviour as described was a potential indication for the prescribed drug in adolescents with intellectual disability, which were dependent on published guidelines and clinical experience.^18–20^ The second step was initiated only if the behaviour was a potential indication. In step 2, the severity of behaviour was assessed to determine whether this was severe enough to warrant pharmacological management, and we determined whether psychological or behavioural approaches had been trialled before medication was used. If the behaviour presented a real likelihood of injury, disability (e.g. blinding) or death, it was judged severe enough to warrant pharmacological treatment. Only if both of these steps were satisfied was the use of medication to manage challenging behaviour considered appropriate. Discrepant decisions were discussed between the two reviewers and consensus was reached. Some study participants transitioned between on-label and off-label, and between appropriate and inappropriate use, within the study period.

### Participant characteristics

Carer questionnaires were completed in May 2007. Information collected included demographic, social and health characteristics including psychopathological status, mobility, communication skills, general health, cause of disability and the presence of epilepsy. Psychopathology was measured using the Developmental Behaviour Checklist-Short Form (DBC-P24).^21^ Total Behaviour Problems Score was applied as an overall measure of behavioural and emotional disturbance, and ‘disturbance’ was identified with the standard cut-off score of 0.48. Participants attended SEUs, SESs or high-needs schools. An SEU is located on the grounds of a mainstream school and students have a range of disabilities; they usually access the mainstream curriculum with specialist teaching and therapy. An SES is a segregated school, and students have significant intellectual disability or multiple disabilities. A high-needs school is a segregated school with staff and facilities for adolescents with profound disability. Specialised staff and facilities allow individualised education and therapy services to be provided. Socioeconomic status was measured at the postcode level, using the Australian Bureau of Statistics Socio-Economic Index for Areas, a measure of relative disadvantage,^22^ and was categorised into thirds.

### Analysis

Summary statistics were presented as median (25th–75th percentile) or frequency (percentage). Proportions were calculated for participant characteristics and medication use. On-label, off-label, clinically appropriate and inappropriate use of psychotropic medications and their subclasses were tabulated. The main on-label, off-label, appropriate and inappropriate use scenarios for psychotropic medications were presented. Analysis was performed with Stata statistical software, version 15 for Windows (StataCorp, TX, USA).

## Results

### Participant characteristics

Medical notes were obtained for 432 adolescents, of whom 429 were followed up for at least 3.5 years and included in our study. The median study time for the 429 participants was 4.2 years (25th–75th percentile: 3.9–4.3 years) (Table 1). Sleep and affective disorders were diagnosed in 38 (8.9%), and 27 (6.3%) of participants, respectively. There were 114 (26.6%) participants recorded as exhibiting one or more challenging behaviours on at least one occasion during the study period.

**Table 1:** Participant characteristics (*N*_participant_ = 429)

	Participant, *N* (%)
Total	429 (100.0)
Female	197 (45.9)
Age (years) on 1 January 2006	
10–13	113 (26.3)
14–16	272 (63.4)
17–20	44 (10.3)
School type	
Special education unit	196 (45.7)
Special education school	227 (52.9)
High support need school	6 (1.4)
Socioeconomic status (thirds)^a^	
Advantaged	187 (43.6)
Medium	140 (32.6)
Disadvantaged	102 (23.8)
General health^a^	
Excellent	113 (26.4)
Very good	145 (33.8)
Good	128 (29.8)
Fair	37 (8.6)
Poor	6 (1.4)
Communication^a^^,^^b^	
Verbal	321 (75.0)
Some verbal with non-verbal aids	59 (13.8)
Non-verbal	48 (11.2)
Mobility^a^	
Walks independently	367 (85.5)
Walks with aids	57 (13.3)
Immobile	5 (1.2)
Cause of intellectual disability^a^	
Down syndrome	65 (15.2)
Other known cause	234 (54.5)
Unknown cause	130 (30.3)
Psychopathology^a^^,^^c^	
No disturbance	129 (30.4)
Disturbance	295 (69.6)
Epilepsy^a^	83 (19.3)
Autism^d^	76 (17.7)
ADHD^d^	61 (14.2)
Mental disorder^d^	
Sleep disorders	38 (8.9)
Affective disorders	27 (6.3)
Anxiety	21 (4.9)
Symptoms and signs involving emotional state	13 (3.0)
Symptoms and signs involving general sensations and perceptions	10 (2.3)
Reaction to severe stress, and adjustment disorders	10 (2.3)
Other mental disorders^e^	12 (2.8)
Challenging behaviour^d^	114 (26.6)

ADHD, attention-deficit hyperactivity disorder.

a. Carer reported.

b. Missing data for one participant.

c. Missing data for five participants.

d. Clinician diagnosed.

e. Other mental disorders includes obsessive–compulsive disorder (four adolescents), specific personality disorders (three adolescents), unspecified nonorganic psychosis (two adolescents), mental and behavioural disorders owing to use of tobacco (one adolescent), schizophrenia (one adolescent), persistent delusional disorders (one adolescent), acute and transient psychotic disorders (one adolescent) and eating disorders (one adolescent).

### Prevalence of psychotropic medications and their subclasses

Over the study period, 107 (24.9%) adolescents were prescribed psychotropic medications at least once (Table 2), with 29.9% of the 107 adolescents concurrently prescribed two or more psychotropic drugs. The most frequently prescribed subclasses were antidepressants (*n* = 51, 47.7%), antipsychotics (*n* = 43, 40.2%) and psychostimulants (*n* = 37, 34.6%).

**Table 2:** Label adherence and appropriateness of use of psychotropic medications with their subclasses between 2006 and 2010

	Psychotropic, *n* (%)	Antidepressants, *n* (%)	Antipsychotics, *n* (%)	Psychostimulants, *n* (%)	Hypnotics/sedatives, *n* (%)	Anxiolytics, *n* (%)	Anti-Parkinsonian drugs, *n* (%)
Number of participants	107 (100.0)	51 (100.0)	43 (100.0)	37 (100.0)	20 (100.0)	14 (100.0)	4 (100.0)
Label adherence							
Always off-label	43 (40.2)	30 (58.8)	20 (46.5)	20 (54.1)	12 (60.0)	11 (78.6)	3 (75.0)
Sometimes off-label	45 (42.0)	10 (19.6)	16 (37.2)	12 (32.4)	3 (15.0)	0 (0.0)	0 (0.0)
Always on-label	19 (17.8)	11 (21.6)	7 (16.3)	5 (13.5)	5 (25.0)	3 (21.4)	1 (25.0)
Appropriateness							
Always inappropriate	53 (49.5)	26 (51.0)	34 (79.1)	21 (56.8)	9 (45.0)	12 (85.7)	2 (50.0)
Sometimes inappropriate	35 (32.7)	14 (27.4)	9 (20.9)	11 (29.7)	4 (20.0)	0 (0.0)	1 (25.0)
Always appropriate	19 (17.8)	11 (21.6)	0 (0.0)	5 (13.5)	7 (35.0)	2 (14.3)	1 (25.0)

### Label adherence and clinical appropriateness of use

Of the 107 adolescents who were prescribed psychotropic medications, 17.8% were prescribed on-label and appropriately throughout the entire study period (Table 2); 21.6% of the 51 adolescents prescribed antidepressants had on-label and appropriate use throughout the study. For psychostimulants, 13.5% of the 37 adolescents had on-label and appropriate use throughout. More than half of the adolescents prescribed antidepressants had off-label (58.8%) or inappropriate (51.0%) use throughout. For adolescents prescribed psychostimulants, 54.1% had off-label use and 56.8% had inappropriate use throughout. Of the 43 adolescents prescribed antipsychotics, all had inappropriate use on at least one occasion, including 79.1% prescribed inappropriately throughout; 16.3% of them were prescribed on-label throughout.

### Scenarios of psychotropic prescribing

During the study period, 461 psychotropic prescriptions were provided to the 107 adolescents. Overlap was common between on-label and appropriate scenarios, and between off-label and inappropriate scenarios (Supplementary Tables 1–4 available at https://doi.org/10.1192/bjo.2020.125). The most common on-label and appropriate use scenario was using psychostimulants (methylphenidate and dexamphetamine, 27 and 11 prescriptions, respectively) for ADHD (Supplementary Tables 1 and 3). Other common on-label and appropriate scenarios were use of escitalopram for anxiety (10 prescriptions) and use of fluoxetine for depression (6 prescriptions).

Challenging behaviour was commonly associated with off-label use or inappropriate use, or both concurrently (Supplementary Tables 2, 4 and 5). In particular, common off-label and inappropriate use included use of amitriptyline and fluoxetine (eight prescriptions, respectively) for aggression and use of methylphenidate (eight prescriptions) for concurrent impulsivity and restlessness without record of ADHD.

Common on-label but inappropriate scenarios were use of risperidone for aggression (15 prescriptions); multiple challenging behaviours, including aggression and obsession (4 prescriptions); impulsivity, agitation and hyperactivity (4 prescriptions); and aggression and self-injury (3 prescriptions) (Supplementary Tables 1 and 4).

## Discussion

### Main findings

One quarter of the cohort of adolescents with intellectual disability living in the community were prescribed psychotropic medication during the study period. Less than a fifth of adolescents prescribed psychotropic medications had on-label and appropriate use throughout the entire study period. Common on-label and appropriate prescriptions included use of psychostimulants to treat ADHD and use of antidepressants to treat anxiety and depression. Off-label or inappropriate use was commonly associated with challenging behaviours. Risperidone was often used on-label but inappropriately, based on expert opinion, for aggression- or impulsivity-associated behaviours.

### Connection with previous research and possible factors contributing to our findings

#### Prevalence of psychotropic medication use

The prevalence of psychotropic medication use identified via medical notes in this study was 24.9%. In our earlier research, a fifth (20.5%) of participants were prescribed psychotropic medications based on cross-sectional, carer-recorded data from the health check booklet from 176 ASK study participants.^13^ The two estimates were similar. The prevalence in this study is consistent with cross-sectional data reported from adolescent out-patients in Turkey (20.4%).^23^

#### Prevalent off-label use

Few studies have considered off-label use of psychotropic medication among people with intellectual disability. The prevalence of off-label use in ASK study participants prescribed psychotropic medications (82.2%) is higher than in two previous studies: a 10-year cohort study of 114 adults receiving service from a single consultant team in the UK, which reported a prevalence of 65.8%; and a cross-sectional study of 56 adult and adolescent in-patients with psychopathology and mild or borderline intellectual disability, who were receiving services from three consultant psychiatrists at a tertiary referral centre in the UK, which reported a prevalence of 68.4%.^5,6^ This difference may be because of the different sample frames.

The frequent off-label use in our study may originate from clinicians’ lack of familiarity or poor adherence to TGA-approved indications. The TGA does not regulate prescribing practice. Approved indications for a medication are often restricted to adult patients with one particular diagnosis, and there is a lack of data for vulnerable groups, including people with intellectual disability, for reasons such as difficulty in recruiting sufficient participants for clinical trials and obtaining participation consent.^5^ Thus, clinicians may prescribe off-label based on other comprehensive information sources, including evidence from other populations, guidelines for prescribing to people with intellectual disability^19,20^ and expert opinion. Clinicians may prioritise clinical appropriateness over label adherence. On-label use is not the sole criterion for clinically appropriate use; phase 4 clinical trials are often encouraged to examine drug safety during sales. Some off-label use may generate more benefits than risks.

#### Prevalent inappropriate use

When deciding the appropriateness of drug use, we focused on diagnosis but, where possible, non-drug approaches and nature of challenging behaviour managed were assessed. Psychotropic medications were commonly used inappropriately in our study. This may be because of inadequate clinician knowledge; inadequate access to specialists for diagnosis or advice; or insufficient coordination between clinicians, staff in disability service, carers and adolescents with intellectual disability. Poor coordination may lead to the misidentification of physical or mental health needs of adolescents. Physical and mental disorders are both potential precipitants of challenging behaviours.^9^ Inappropriate use may be initiated for challenging behaviours when their causes are misidentified. Inappropriate use may occur during a treatment trial before definitive diagnosis is made. Inappropriate use may also be influenced by carers’ or individuals’ preference for medications.

#### Particular use scenarios

In line with previous studies,^4^ inappropriate use of psychotropic medications was common with challenging behaviours. More than a quarter (26.6%) of our study participants were recorded exhibiting challenging behaviours on at least one occasion. Among participants exhibiting challenging behaviours, more than half (53.5%) exhibited multiple challenging behaviours (for example, concurrent aggression and self-injury). The complexity of behaviours may mask mental or physical disorders or hamper diagnosis. Complex behaviours also make the recognition of environmental factors contributing to behaviours difficult; consequently, effective non-drug management may not be applied. Psychotropic medications may be used inappropriately when non-drug approaches are not feasible or are not put in place for another reason.

Although TGA-approved indications for risperidone include aggression and impulsivity in adolescents with intellectual disability, using risperidone to manage these behaviours in our study was determined to be inappropriate after considering whether psychological or behavioural approaches were prioritised or combined with pharmacotherapy, and whether the behaviours were severe enough to warrant pharmacological management.

### Recommendations for improving the use of psychotropic medications in Australia

Psychostimulants for ADHD and antidepressants for anxiety and depression were often prescribed on-label and appropriately. However, more attention is required to the use of psychotropic drugs for challenging behaviours. More high-quality, randomised controlled trials to examine both on-label and off-label use of psychotropic medications for challenging behaviours are needed. Severity and persistence of challenging behaviours should be determined, and pragmatic interventions combining medication and positive behaviour support should be incorporated in trials.

To reduce or eliminate inappropriate use of psychotropic medications, the programme of stopping overmedication of people with a learning disability, autism or both (STOMP) has been launched in the UK, incorporating regular check of prescriptions and cooperation among health professionals, carers and other support staff in making drug-related decisions.^24^ In the Netherlands, studies have been conducted to investigate psychotropic discontinuation; evidence created should be operationalised to reduce reliance on antipsychotics for behaviour management. Factors which may influence psychotropic discontinuation were investigated from a broad range of perspectives, including individual characteristics; attitude, knowledge and beliefs of support staff; and cooperation among clinicians, support staff and carers.^25–27^ Effects of discontinuation on behaviours have been examined by a trial.^28^ The STOMP programme in the UK and studies in the Netherland may provide evidence for reducing inappropriate use of psychotropic medications in Australia.

To ensure safe and appropriate psychotropic prescribing practice in Australia, assessment, management and follow-up of challenging behaviour are important. A multidisciplinary group of health professionals should be involved in assessment.^29^ Medical conditions, the social environment and other environmental factors should be assessed, taken into account and modified when feasible and appropriate.^30^

Better coordination is needed among different support areas (for example, health, disability, education and family support) to aid appropriate use of psychotropic medications for challenging behaviours, and follow-up of treatment. The introduction of the National Disability Insurance Scheme (NDIS) has the potential to promote coordination. The scheme was legislated in 2013 and went into full operation in 2020.^31^ NDIS is a welfare support scheme of the Australian Government that funds costs associated with disability, which covers the funds for therapeutic supports, including behaviour support, daily personal activities, mobility and development of daily life skills.^32^ Local area coordinators were employed to work in different communities of Australia to help people with intellectual disability understand and access the NDIS.^33^ The coordinators also work with eligible participants to create, use and review individualised plans and make communities more accessible and inclusive for participants.^33^ In particular, the NDIS helps participants realise their plans by connecting them to community supports,^32^ and provides information for the linkage between the NDIS and other government services such as health, education and disability services.^34^ The NDIS Quality and Safeguards Commission was established to regulate NDIS service providers, and to ensure the consistency, safety and quality of the service.^35^

The Positive Behaviour Support Capability Framework has been created by the NDIS Quality and Safeguards Commission to strengthen the safeguards for people receiving behaviour support.^10^ In this Framework, knowledge and skill requirements for NDIS service providers are stipulated to ensure evidence-based positive behaviour support is in place to reduce restrictive practices. In some cases, using psychotropic medications as chemical restraints for challenging behaviours are inappropriate, and may be defined as a restrictive practice.

### Strengths and limitations

Our study is the first to investigate the proportion of inappropriate use of psychotropic medications in adolescents with intellectual disability. It is also the first to examine label adherence and clinical appropriateness of psychotropic medication use. Participants are similar in age and socioeconomic status to the population of Queensland school adolescents from which they are drawn, although there is a slightly greater proportion of females in our sample. In the ASK study, drop-out was unrelated to carer or participant characteristics.^36^ Participants accessed medical services from clinicians located in the communities of participants’ residence. Clinical prescribing practices are unlikely to have changed much from 2010 to the present because of a limited focus on psychotropic medication use in people with intellectual disability and limited development of specialist capacity in intellectual disability healthcare in Australia. Consequently, these results are likely to have good external validity. Limitations include that data were extracted from medical notes, which are designed as a record for individual clinicians and can vary widely in completeness. The reporting of challenging behaviour depended on the availability of adequate records of behaviour in medical notes, and may be subject to the bias of carers. It is possible the prevalence of inappropriate use of psychotropic medications in challenging behaviours was overestimated as non-drug management, and significant behavioural severity may have occurred before the study period, leading us to misclassify appropriate use as inappropriate.

## Data Availability

All authors have access to the original study data. The data that support the findings of this study are available from the corresponding author, D.H., upon reasonable request.
