# Supplementary material for: Appropriateness of psychotropic medication use in a cohort of adolescents with intellectual disability in Queensland, Australia
Source: BJPsych Open. 2020 Nov 17;6(6):e142. doi: 10.1192/bjo.2020.125 (PMC7745239; doi:10.1192/bjo.2020.125)
Supplement: Supplementary file 1 [file bjosup.zip › S2056472420001258sup001.doc]

**Supplementary Material 1**

**Table S1. On-label use of psychotropic drugs between 2006 and 2010**

| **Indication** | **Drug (number of prescriptions n_p_, number of adolescents n_a_)** |
| --- | --- |
| Challenging behaviour |  |
| Aggression | risperidone (n_p_ =15, n_a_ =10) |
| Aggression, self-injury, agitation | periciazine (n_p_=5, n_a_=1) |
| Aggression and obsession | risperidone (n_p_=4, n_a_=3) |
| Impulsivity, agitation, and hyperactivity | risperidone (n_p_=4, n_a_ =1); dexamphetamine (n_p_=4, n_a_=1) |
| Aggression and self-injury | risperidone (n_p_=3, n_a_=2) |
| Attention deficit hyperactivity disorder | methylphenidate (n_p_=27, n_a_=11); dexamphetamine (n_p_=11, n_a_=7) |
| Anxiety | escitalopram (n_p_=10, n_a_=2); |
| Depression | fluoxetine (n_p_=6, n_a_=3); citalopram (n_p_=4, n_a_=1); escitalopram (n_p_=3, n_a_=3); |
| Schizophrenia related psychosis | olanzapine (n_p_=5, n_a_=1) |
| Sleep disorders | melatonin (n_p_=3, n_a_=3) |
| Obsessive-compulsive disorder | paroxetine (n_p_=3, n_a_=2) |

Prescriptions (n≥3) were present.

**Table S2. Off-label use of psychotropic drugs between 2006 and 2010**

| **Indication** | **Drug (number of prescriptions n_p_, number of adolescents n_a_)** |
| --- | --- |
| Challenging behaviour |  |
| Aggression | amitriptyline (n_p_=8, n_a_=4); fluoxetine (n_p_=8, n_a_=2); methylphenidate (n_p_=7, n_a_=6);  olanzapine (n_p_=5, n_a_=3); quetiapine (n_p_=3, n_a_=3); dexamphetamine (n_p_=3, n_a_=2);  escitalopram (n_p_=3, n_a_=2); |
| Impulsivity, restlessness (without records of associated ADHD) | methylphenidate (n_p_=8, n_a_=4) |
| Aggression, self-injury | methylphenidate (n_p_=3, n_a_=2) |
| Autism (without records of associated behavioural disorders) | risperidone (n_p_=11, n_a_=7); methylphenidate (n_p_=6, n_a_=5) |
| Anxiety | fluoxetine (n_p_=9, n_a_=3); fluvoxamine (n_p_=5, n_a_=3); risperidone (n_p_=5, n_a_=3) |
| Epilepsy | fluvoxamine (n_p_=6, n_a_=1); trihexyphenidyl (n_p_=5, n_a_=2); midazolam (n_p_=4, n_a_=3);  nitrazepam (n_p_=4, n_a_=3); risperidone (n_p_=3, n_a_=3); periciazine (n_p_=3, n_a_=2);  diazepam (n_p_=3, n_a_=2); quetiapine (n_p_=3, n_a_=1) |
| Eating disorders | lithium (n_p_=4, n_a_=1); quetiapine (n_p_=4, n_a_=1); clomipramine (n_p_=3, n_a_=1) |
| Tic disorders | clomipramine (n_p_=3, n_a_=1) |

Prescriptions (n≥3) were present. ADHD: attention deficit hyperactivity disorder.

**Table S3. Appropriate use of psychotropic drugs between 2006 and 2010**

| **Indication** | **Drug (number of prescriptions n_p_, number of adolescents n_a_)** |
| --- | --- |
| Challenging behaviour |  |
| Aggression | olanzapine (n_p_=3, n_a_=1) |
| Attention deficit hyperactivity disorder | methylphenidate (n_p_=27, n_a_=11); dexamphetamine (n_p_=11, n_a_=7) |
| Anxiety | escitalopram (n_p_=10, n_a_=2); fluoxetine (n_p_=9, n_a_=3); fluvoxamine (n_p_=5, n_a_=3) |
| Depression | fluoxetine (n_p_=6, n_a_=3); escitalopram (n_p_=3, n_a_=3); citalopram (n_p_=4, n_a_=1) |
| Schizophrenia related psychosis | olanzapine (n_p_=5, n_a_=1) |
| Epilepsy | midazolam (n_p_=4, n_a_=3) |
| Sleep disorders | melatonin (n_p_=3, n_a_=3) |
| Obsessive-compulsive disorder | paroxetine (n_p_=3, n_a_=2) |

Prescriptions (n≥3) were present.

**Table S4. Inappropriate use of psychotropic drugs between 2006 and 2010**

| **Indication** | **Drug (number of prescriptions n_p_, number of adolescents n_a_)** |
| --- | --- |
| Challenging behaviour |  |
| Aggression | risperidone (n_p_=15, n_a_=10); amitriptyline (n_p_=8, n_a_=4); fluoxetine (n_p_=8, n_a_=2)  methylphenidate (n_p_=7, n_a_=6); quetiapine (n_p_=3, n_a_=3); dexamphetamine (n_p_=3, n_a_=2); escitalopram (n_p_=3, n_a_=2) |
| Impulsivity, restlessness (without records of associated ADHD) | methylphenidate (n_p_=8, n_a_=4) |
| Aggression, self-injury, agitation | periciazine (n_p_=5, n_a_=1) |
| Aggression, obsession | risperidone (n_p_=4, n_a_=3) |
| Impulsivity, agitation, hyperactivity | risperidone (n_p_=4, n_a_=1); dexamphetamine (n_p_=4, n_a_=1) |
| Aggression, self-injury | risperidone (n_p_=3, n_a_=2); methylphenidate (n_p_=3, n_a_=2) |
| Autism (without records of associated behavioural disorders) | risperidone (n_p_=11, n_a_=7); methylphenidate (n_p_=6, n_a_=5) |
| Epilepsy | fluvoxamine (n_p_=6, n_a_=1); trihexyphenidyl (n_p_=5, n_a_=2); nitrazepam (n_p_=4, n_a_=3);  risperidone (n_p_=3, n_a_=3); periciazine (n_p_=3, n_a_=2); diazepam (n_p_=3, n_a_=2);  quetiapine (n_p_=3, n_a_=1) |
| Anxiety | risperidone (n_p_=5, n_a_=3) |
| Eating disorder | lithium (n_p_=4, n_a_=1); quetiapine (n_p_=4, n_a_=1); clomipramine (n_p_=3, n_a_=1) |
| Tic disorders | clomipramine (n_p_=3, n_a_=1) |

Prescriptions (n≥3) were present. ADHD: attention deficit hyperactivity disorder.

**Table S5. Determining appropriateness of psychotropic medication use for challenging behaviours in particular occasions**

| **Diagnosis** | **Medication** | **Clinical appropriateness** |
| --- | --- | --- |
| Challenging behaviour |  |  |
| Aggression | alprazolam | inappropriate |
|  | amitriptyline | inappropriate |
|  | atomoxetine | inappropriate |
|  | benzodiazepine | inappropriate |
|  | clomipramine | inappropriate |
|  | dexamphetamine | inappropriate |
|  | escitalopram | inappropriate |
|  | fluoxetine | inappropriate |
|  | fluvoxamine | inappropriate |
|  | haloperidol | inappropriate |
|  | melatonin | inappropriate |
|  | methylphenidate | inappropriate |
|  | olanzapine | appropriate |
|  | periciazine | inappropriate |
|  | quetiapine | inappropriate |
|  | reboxetine | inappropriate |
|  | risperidone | inappropriate |
|  | sertraline | inappropriate |
| Self-injury | amitriptyline | inappropriate |
|  | atomoxetine | inappropriate |
|  | escitalopram | inappropriate |
|  | paroxetine | inappropriate |
|  | sertraline | inappropriate |
| Impulsivity | amitriptyline | inappropriate |
|  | dexamphetamine | inappropriate |
|  | haloperidol | inappropriate |
|  | methylphenidate | inappropriate |
|  | risperidone | inappropriate |
| Withdrawal | risperidone | inappropriate |
|  | haloperidol | inappropriate |
|  | methylphenidate | inappropriate |
|  | amitriptyline | inappropriate |
| Obsession | clobazam | inappropriate |
| Non-cooperation | melatonin | inappropriate |
| Aggression, self-injury | diazepam | inappropriate |
|  | fluoxetine | inappropriate |
|  | haloperidol | inappropriate |
|  | methylphenidate | inappropriate |
|  | risperidone | inappropriate |
| Aggression, impulsivity | escitalopram | inappropriate |
|  | risperidone | inappropriate |
|  | quetiapine | inappropriate |
| Aggression, property destruction | risperidone | inappropriate |
|  | zopiclone | inappropriate |
| Aggression, obsession | atomoxetine | inappropriate |
|  | fluvoxamine | inappropriate |
|  | risperidone | inappropriate |
| Aggression, withdrawal | amitriptyline | inappropriate |
|  | atomoxetine | inappropriate |
|  | methylphenidate | inappropriate |
| Aggression, non-cooperation | atomoxetine | inappropriate |
|  | dexamphetamine | inappropriate |
|  | methylphenidate | inappropriate |
|  | risperidone | inappropriate |
| Aggression, absence | aripiprazole | inappropriate |
|  | imipramine | inappropriate |
| Aggression, laughing inappropriately | clomipramine | inappropriate |
|  | periciazine | inappropriate |
| Aggression, sexually inappropriate behaviour | dexamphetamine | inappropriate |
|  | risperidone | inappropriate |
| Aggression, poor concentration span | methylphenidate | inappropriate |
| Aggression, screaming | escitalopram | inappropriate |
| Self-injury, agitation | amitriptyline | inappropriate |
|  | fluoxetine | inappropriate |
| Agitation, impulsivity | sertraline | inappropriate |
| Agitation, poor concentration span | sertraline | inappropriate |
| Agitation, property destruction | citalopram | inappropriate |
| Agitation, screaming | sertraline | inappropriate |
| Agitation, non-compliance with drugs | escitalopram | inappropriate |
| Impulsivity, restlessness | methylphenidate | inappropriate |
|  | risperidone | inappropriate |
|  | dexamphetamine | inappropriate |
| Impulsivity, non-compliance with drugs | methylphenidate | inappropriate |
| Aggression, agitation, property destruction | duodopa | inappropriate |
|  | methylphenidate | inappropriate |
| Aggression, agitation, self-injury | periciazine | inappropriate |
|  | risperidone | inappropriate |
| Aggression, agitation, impulsivity | methylphenidate | inappropriate |
| Aggression, agitation, non-compliance with drugs | methylphenidate | inappropriate |
|  | risperidone | inappropriate |
| Aggression, agitation, sexually inappropriate behaviour | flunitrazepam | inappropriate |
|  | fluoxetine | inappropriate |
| Aggression, withdrawal, fixated and repetitive behaviour | dexamphetamine | inappropriate |
|  | olanzapine | inappropriate |
| Aggression, impulsivity, screaming | risperidone | inappropriate |
| Aggression, absence, stalking people | olanzapine | appropriate |
| Impulsivity, agitation, hyperactivity | dexamphetamine | inappropriate |
|  | risperidone | inappropriate |
| Agitation, obsession, property destruction | fluvoxamine | inappropriate |
|  | sertraline | inappropriate |
| Non-specified behaviour | amitriptyline | inappropriate |
|  | dexamphetamine | inappropriate |
|  | imipramine | inappropriate |
|  | reboxetine | inappropriate |
|  | risperidone | inappropriate |
|  | paroxetine | inappropriate |
|  | periciazine | inappropriate |
|  | quetiapine | inappropriate |

**Table S6. The association between psychopathology and use of psychotropic medications (N=429 participants)**

|  | **Participant**  **N** | **Psychotropic medications**  **n (%)** | **Odds ratio**  **(95% CI)** |
| --- | --- | --- | --- |
| Psychopathology ^†^ |  |  |  |
| No disturbance | 129 | 12 (9.3) | 1.0 |
| Minor disturbance | 114 | 28 (24.6) | 3.2 (1.5-6.6) ^*^ |
| Major disturbance | 181 | 66 (36.5) | 5.6 (2.9-10.9) ^*^ |

CI, confidence interval

^*^*P*<0.05

^†^5 participants missing

**Table S7. The association between psychopathology and label-adherence of psychotropic medication use (N=107 participants)**

|  | **Participants prescribed psychotropic drugs**  **N** | **Always**  **on-label^‡^**  **n (%)** | **Sometimes off-label**  **n (%)** | **Odds ratio (95%CI)** | **Always off-label**  **n (%)** | **Odds ratio (95%CI)** |
| --- | --- | --- | --- | --- | --- | --- |
| Psychopathology^†^ |  |  |  |  |  |  |
| No disturbance | 12 | 4 (33.3) | 2 (16.7) | 1.0 | 6 (50.0) | 1.0 |
| Minor disturbance | 28 | 10 (35.6) | 9 (32.2) | 1.8 (0.3-12.3) | 9 (32.2) | 0.6 (0.1-2.8) |
| Major disturbance | 66 | 5 (7.6) | 34 (51.5) | 13.6 (2.0-94.6)^*^ | 27 (40.9) | 3.6 (0.7-17.6) |

CI, confidence interval

^*^*P*<0.05

^†^ 1 participant missing

^‡^ Base outcome

**Table S8. The association between psychopathology and appropriateness of psychotropic medication use (N=107 participants)**

|  | **Participants prescribed psychotropic drugs**  **N** | **Always appropriate^‡^**  **n (%)** | **Sometimes inappropriate**  **n (%)** | **Odds Ratio (95%CI)** | **Always inappropriate**  **n (%)** | **Odds Ratio (95%CI)** |
| --- | --- | --- | --- | --- | --- | --- |
| Psychopathology^†^ |  |  |  |  |  |  |
| No disturbance | 12 | 4 (33.3) | 1 (8.3) | 1.0 | 7 (58.4) | 1.0 |
| Minor disturbance | 28 | 10 (35.7) | 10 (35.7) | 4.0 (0.4-42.4) | 8 (28.6) | 0.5 (0.1-2.1) |
| Major disturbance | 66 | 5 (7.6) | 24 (36.4) | 19.2 (1.8-210.3)* | 37 (56.0) | 4.2 (0.9-19.8) |

CI, confidence interval

^*^*P*<0.05

^†^ 1 participant missing

^‡^ Base outcome
